# Chemical Evidence for Potent Xanthine Oxidase Inhibitory Activity of Ethyl Acetate Extract of *Citrus aurantium* L. Dried Immature Fruits

**DOI:** 10.3390/molecules21030302

**Published:** 2016-03-02

**Authors:** Kun Liu, Wei Wang, Bing-Hua Guo, Hua Gao, Yang Liu, Xiao-Hong Liu, Hui-Li Yao, Kun Cheng

**Affiliations:** School of Pharmacy, Qingdao University, Qingdao 266021, Shandong, China; kunliu62@126.com (K.L.); guobinghua1214@126.com (B.-H.G.); gaohuaqy@126.com (H.G.); buckuper@163.com (Y.L.); liuxiaohong1043@163.com (X.-H.L.); xyhuili@126.com (H.-L.Y.); chengkun1990518@163.com (K.C.)

**Keywords:** *Citrus*, xanthine oxidase, aurantii fructus immaturus, hesperetin

## Abstract

Xanthine oxidase is a key enzyme which can catalyze hypoxanthine and xanthine to uric acid causing hyperuricemia in humans. Xanthine oxidase inhibitory activities of 24 organic extracts of four species belonging to *Citrus* genus of the family Rutaceae were assayed *in vitro*. Since the ethyl acetate extract of *C*. *aurantium* dried immature fruits showed the highest xanthine oxidase inhibitory activity, chemical evidence for the potent inhibitory activity was clarified on the basis of structure identification of the active constituents. Five flavanones and two polymethoxyflavones were isolated and evaluated for inhibitory activity against xanthine oxidase *in vitro*. Of the compounds, hesperetin showed more potent inhibitory activity with an IC_50_ value of 16.48 μM. For the first time, this study provides a rational basis for the use of *C*. *aurantium* dried immature fruits against hyperuricemia.

## 1. Introduction

Epidemiological data show a rise in the prevalence of gout that is potentially attributable to shifts in diet and lifestyle and increased longevity [1]. Gout is a disorder of purine metabolism and results from urate crystal deposition in and around the joints caused by longstanding hyperuricemia. Xanthine oxidase (XO), an enzyme present in significant concentrations in the gastrointestinal and liver, is responsible for the metabolism of hypoxanthine and xanthine to uric acid in the purine catabolic pathway. Accordingly, the use of the XO inhibitor that blocks the synthesis of uric acid in the body should be one of the therapeutic approaches for the treatment of hyperuricemia and chronic gout [2]. Some synthetic XO inhibitors, such as allopurinol, febuxostat, and Y-700, have shown good efficacies against hyperuricemia and chronic gout [3,4,5]. However, they also cause side effects, such as allergic and hypersensitivity reactions, renal failure, skin rash, gastrointestinal distress, and enhancement of 6-mercaptopurine toxicity [6]. As a result of increasing consumer preference toward more natural and healthier products, scientific research has begun to focus on the screen of natural compounds as possible XO inhibitors [7,8,9,10].

*Citrus* is a genus of small trees or large shrubs that comprises more than 20 species widely distributed throughout the worldwide tropics and subtropics, the original range of this genus can be traced to Southeast Asia and India. The main uses of *Citrus* in food industries include fresh juice or *Citrus*-based drinks. Additionally, *Citrus* fruits are well-known sources of flavonoids, terpenes, coumarins, and alkaloids with potential health-promoting properties related to their anticancer, antioxidant, anti-inflammatory, antimicrobial, hypolipidemic, hepatoprotective, neuroprotective, antiedematogenic, and cardiovascular activities [11,12]. As a continuation of our screening program relating to plants with XO inhibitory activity [13,14], the present work describes, for the first time, a systematic *in vitro* XO inhibitory assay of 24 organic extracts of four species belonging to *Citrus* genus. The results revealed that the ethyl acetate extract of *C*. *aurantium* dried immature fruits has potent XO inhibitory activity. However, it is still unclear which compounds are the active ingredients in the extract, and the contributions of the main identified compounds to the XO inhibitory activity of the extract are unknown. The present study seeks to investigate the potent XO inhibitors from the ethyl acetate extract of *C*. *aurantium* dried immature fruits. The study benefits would explain and support application of the ethyl acetate extract of *C*. *aurantium* dried immature fruits as a complementary medicine for the prevention and treatment of hyperuricemia.

## 2. Results and Discussion

### 2.1. XO Inhibitory Activity of the Extracts

XO belongs to molybdenum hydroxylase superfamily and consists of two identical subunits of 145 kDa, which participates in purine degradation. During these reactions, it uses molecular oxygen as the electron acceptor, thereby resulting in production of superoxide anion radical and hydrogen peroxide [15]. The antioxidant property of extract from *Citrus* genus are well documented, but considerably less work has been perform regarding the inhibition of XO [16,17,18]. As extending studies, the XO inhibitory activities of 24 organic extracts of four species belonging to *Citrus* genus were evaluated. The results of the assays are listed in Table 1. The extracts were prepared with petroleum ether (A), ethyl acetate (B), butanol (C), and ethanol–water (75:25, D) from selected plant organs. Although there is no generally accepted threshold for efficacy in our experiment, XO inhibitory effects of <10% were considered irrelevant and are, therefore, not presented in Table 1. A total of nine extracts demonstrated substantial XO inhibitory activity (>30%) at 200μg/mL, while *C*. *aurantium* dried immature fruit extracts exhibited much higher inhibitions than those of other selected plant same solvent extracts (*p* < 0.05). In the case of *C*. *aurantium* dried immature fruits, ethyl acetate extract showed the highest inhibition of XO activity than did petroleum ether, butanol, and ethanol–water (75:25) extracts (*p* < 0.05). Percent inhibition was calculated to be 89.24% ± 0.69% for allopurinol, a clinical XO inhibitory drug, at 1 μg/mL.

### 2.2. Identification of the Constituents of the Most Active Extract

**Figure 1:**
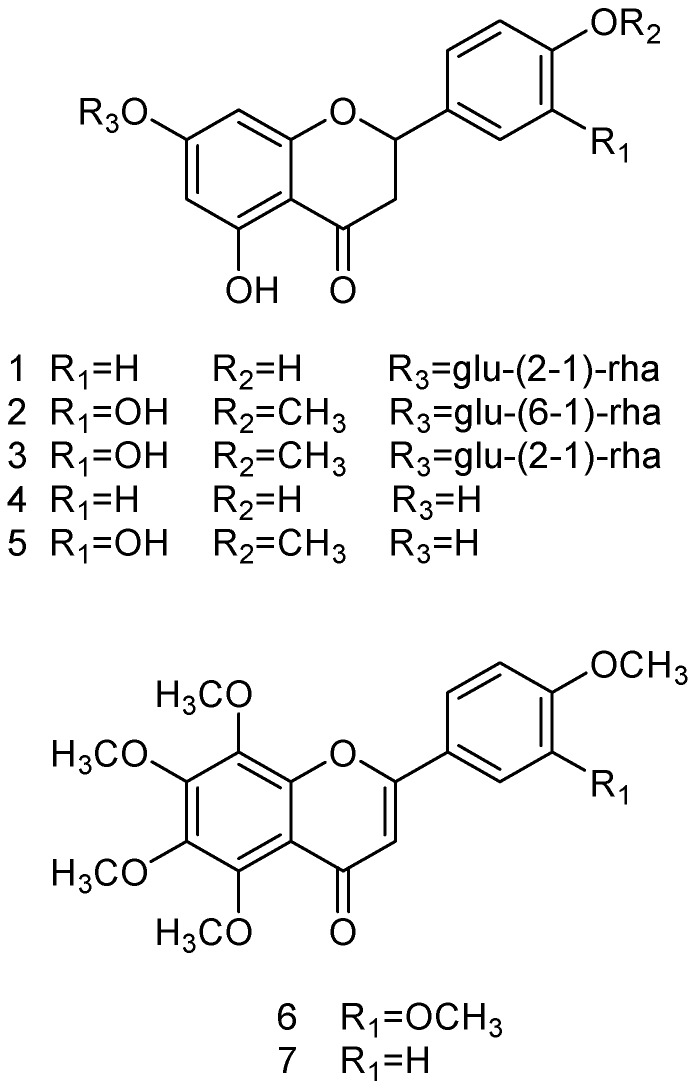
Structures of the compounds identified from the ethyl acetate extract of *C*. *aurantium* dried immature fruits: naringin (**1**); hesperidin (**2**); neohesperidin (**3**); naringenin (**4**); hesperetin (**5**); nobiletin (**6**); and tangeretin (**7**).

**Figure 2:**
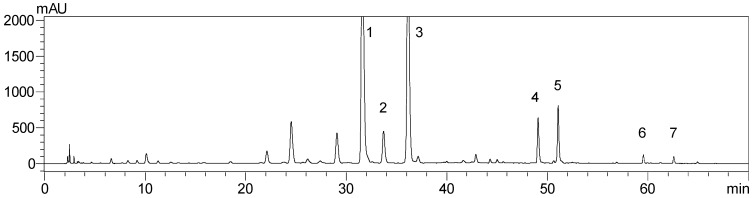
HPLC profile of the ethyl acetate extract of *C*. *aurantium* dried immature fruits and compound-identified peaks: naringin (**1**); hesperidin (**2**); neohesperidin (**3**); naringenin (**4**); hesperetin (**5**); nobiletin (**6**); and tangeretin (**7**).

The ethyl acetate extract of *C*. *aurantium* dried immature fruits was fractionated by RP-18 reversed-phase silica gel column chromatography into six fractions. The active fractions were further purified by preparative HPLC to isolated seven compounds. Spectroscopic analysis and comparison with literature values were used to identify the structure of isolated compounds as naringin (**1**) [19,20], hesperidin (**2**), neohesperidin (**3**) [19,21], naringenin (**4**) [19,22], hesperetin (**5**) [19,23], nobiletin (**6**), and tangeretin (**7**) [24]. The confirming spectroscopic data for naringin are as follows: ESI-MS at *m/z* 581 [M + H]^+^; ^1^H-NMR (500 MHz, DMSO-*d*_6_) 7.32 (2H, d, *J* = 8.6 Hz, H-2′, 6′), 6.80 (2H, d, *J* = 8.4 Hz, H-3′,5′), 6.12 (1H, d, *J* = 2.2 Hz, H-6), 6.11 (1H, d, *J* = 2.2 Hz, H-8), 5.52 (1H, dd, *J* = 13.2, 2.8 Hz, H-2), 5.14 (1H, d, *J* = 7.4 Hz, H-1′′), 5.10 (1H, d, *J* = 2.2 Hz, H-1′′′), 2.72 (1H, dd, *J* = 17.1, 2.8 Hz, H-3_ax_), 1.15 (3H, d, *J* = 6.2 Hz, H-6′′′); ^13^C-NMR (125 MHz, DMSO-*d*_6_) 197.3 (C-4), 164.8 (C-7), 162.9 (C-5), 162.8 (C-9), 157.8 (C-4′), 146.8 (C-5), 130.5 (C-6), 128.6 (C-1′), 128.5 (C-2′, 6′), 115.2 (C-3′, 5′), 103.3 (C-10), 100.4 (C-1′′′), 97.4 (C-1′′), 96.3 (C-6), 95.1 (C-8), 78.8 (C-2), 77.1 (C-2′′), 76.9 (C-3′′), 76.2 (C-5′′), 71.8 (C-4′′′), 70.4 (C-3′′′), 70.3 (C-2′′′), 69.6 (C-4′′), 68.2 (C-5′′′), 60.4 (C-6′′), 42.1 (C-3), 18.0 (C-6′′′). The confirming spectroscopic data for hesperidin are as follows: ESI-MS at *m/z* 611 [M + H]^+^; ^1^H-NMR (500 MHz, DMSO-*d*_6_) 6.89–6.95 (3H, overlap, H-2′, 5′, 6′), 6.15 (1H, d, *J* = 2.4 Hz, H-6), 6.12 (1H, d, *J* = 2.4 Hz, H-8), 5.50 (1H, dd, *J* = 12.2, 3.2 Hz, H-2), 4.99 (1H, d, *J* = 7.4 Hz, H-1′′), 4.52 (1H, br s, H-1′′′), 3.77 (3H, s, OCH_3_), 2.76 (1H, dd, *J* = 17.1, 3.2 Hz, H-3_ax_), 1.08 (3H, d, *J* = 6.2 Hz, H-6′′′); ^13^C-NMR (125 MHz, DMSO-*d*_6_) 196.9 (C-4), 165.1 (C-7), 163.0 (C-5), 162.5 (C-9), 147.9 (C-4′), 146.4 (C-3′), 131.0 (C-1′), 117.9 (C-6′), 114.1 (C-5′), 112.1 (C-2′), 103.3 (C-10), 100.6 (C-1′′′), 99.4 (C-1′′), 96.3 (C-6), 95.6 (C-8), 78.4, 78.3 (C-2), 76.2 (C-3′′), 75.5 (C-5′′), 73.0 (C-2′′), 72.0 (C-4′′′), 70.7 (C-3′′′), 70.2 (C-2′′′), 69.6 (C-4′′), 68.3 (C-5′′′), 66.0 (C-6′′), 56.0 (OCH_3_), 42.0 (C-3), 17.8 (C-6′′′). The confirming spectroscopic data for neohesperidin are as follows: ESI-MS at *m/z* 611 [M + H]^+^; ^1^H-NMR (500 MHz, DMSO-*d*_6_, δ) 6.94 (1H, d, *J* = 8.3 Hz, H-5′), 6.93 (1H, d, *J* = 2.0 Hz, H-2′), 6.88 (1H, dd, *J* = 8.3, 2.0 Hz, H-6′), 6.12 (1H, d, *J* = 2.2 Hz, H-6), 6.09 (1H, d, *J* = 2.2 Hz, H-8), 5.51 (1H, dd, *J* = 12.2, 3.0 Hz, H-2), 5.12 (1H, d, *J* = 7.6 Hz, H-1′′), 5.11 (1H, d, *J* = 2.3 Hz, H-1′′′), 3.78 (3H, s, OCH_3_), 2.77 (1H, dd, *J* = 17.2, 3.0 Hz, H-3_ax_), 1.16 (3H, d, *J* = 6.2 Hz, H-6′′′); ^13^C-NMR (125 MHz, DMSO-*d*_6_, δ) 196.9 (C-4), 164.8 (C-7), 162.9 (C-5), 162.5 (C-9), 148.0 (C-4′), 146.5 (C-3′), 130.9 (C-1′), 117.7 (C-6′), 114.1 (C-5′), 112.0 (C-2′), 103.3 (C-10), 100.3 (C-1′′′), 97.4 (C-1′′), 96.2 (C-6), 95.1 (C-8), 78.4 (C-2), 77.1 (C-2′′), 76.9 (C-3′′), 76.0 (C-5′′), 71.8 (C-4′′′), 70.4 (C-3′′′), 70.3 (C-2′′′), 69.6 (C-4′′), 68.2 (C-5′′′), 60.4 (C-6′′), 55.7 (OCH_3_), 42.1 (C-3), 18.0 (C-6′′′). The confirming spectroscopic data for naringenin are as follows: ESI-MS at *m/z* 271 [M − H]^−^; ^1^H-NMR (500 MHz, DMSO-*d*_6_, δ) 7.31 (2H, d, *J* = 8.5 Hz, H-2′, 6′), 6.80 (2H, dd, *J* = 8.5 Hz, H-3′, 6′), 5.88 (2H, s, H-6, 8), 5.44 (1H, dd, *J* = 12.8, 2.9 Hz, H-2), 3.26 (1H, dd, *J* = 17.1, 12.8 Hz, H-3_eq_), 2.68 (1H, dd, *J* = 17.1, 2.9 Hz, H-3_ax_); ^13^C-NMR (125 MHz, DMSO-*d*_6_, δ) 196.3 (C-4), 166.6 (C-7), 163.4 (C-5), 162.9 (C-9), 157.7 (C-4′), 128.8 (C-1′), 128.2 (C-2′, 6′), 115.1 (C-3′, 5′), 101.7 (C-10), 95.8 (C-6), 94.9 (C-8), 78.4 (C-2), 41.9 (C-3). The confirming spectroscopic data for hesperetin are as follows: ESI-MS at *m/z* 301 [M − H]^−^; ^1^H-NMR (500 MHz, DMSO-*d*_6_, δ) 6.93 (1H, d, *J* = 8.2 Hz, H-5′), 6.92 (1H, br s, H-2′), 6.87 (1H, dd, *J* = 8.2, 2.0 Hz, H-6′), 5.90 (1H, d, *J* = 2.0 Hz, H-8), 5.88 (1H, d, *J* = 2.0 Hz, H-6), 5.43 (1H, dd, *J* = 12.3, 3.0 Hz, H-2), 3.78 (3H, s, OCH_3_), 3.19 (1H, dd, *J* = 17.1, 12.3 Hz, H-3_eq_), 2.71 (1H, dd, *J* = 17.1, 3.0 Hz, H-3_ax_); ^13^C-NMR (125 MHz, DMSO-*d*_6_, δ) 196.1 (C-4), 166.6 (C-7), 163.4 (C-5), 162.8 (C-9), 147.8 (C-4′), 146.4 (C-3′), 131.1 (C-1′), 117.6 (C-6′), 114.0 (C-5′), 112.0 (C-2′), 101.8 (C-10), 95.8 (C-6), 95.0 (C-8), 78.2 (C-2), 55.7 (OCH_3_), 42.0 (C-3). The confirming spectroscopic data for nobiletin are as follows: ESI-MS at *m/z* 403 [M + H]^+^; ^1^H-NMR (500 MHz, DMSO-*d*_6_, δ) 7.65 (1H, dd, *J* = 8.4, 2.0 Hz, H-6′), 7.55 (1H, d, *J* = 2.0 Hz, H-2′), 7.16 (1H, d, *J* = 8.4 Hz, H-5′), 6.86 (1H, s, H-3), 4.03 (3H, s, 7-OCH_3_), 3.98 (3H, s, 5-OCH_3_), 3.88 (3H, s, 3′-OCH_3_), 3.85 (3H, s, 4′-OCH_3_), 3.84 (3H, s, 6-OCH_3_), 3.79 (3H, s, 8-OCH_3_); ^13^C-NMR (125 MHz, DMSO-*d*_6_, δ) 175.8 (C-4), 160.2 (C-2), 151.7 (C-4′), 150.9 (C-7), 149.0 (C-3′), 147.5 (C-8), 147.1 (C-9), 143.5 (C-5), 137.6 (C-6), 123.1 (C-1′), 119.3 (C-6′), 114.6 (C-10), 11.8 (C-5′), 108.9 (C-2′), 106.3 (C-3), 61.8 (5-OCH_3_), 61.7 (7-OCH_3_), 61.4 (3′-OCH_3_), 61.3 (4′-OCH_3_), 55.7 (8-OCH_3_), 55.6 (6-OCH_3_). The confirming spectroscopic data for tangeretin are as follows: ESI-MS at *m/z* 373 [M + H]^+^; ^1^H-NMR (500 MHz, DMSO-*d*_6_, δ) 8.00 (2H, d, *J* = 8.9 Hz, H-2′, 6′), 7.14 (2H, dd, *J* = 8.9 Hz, H-3′, 6′), 6.76 (1H, s, H-3), 4.02 (3H, s, 7-OCH_3_), 3.97 (3H, s, 5-OCH_3_), 3.86 (3H, s, 4′-OCH_3_), 3.84 (3H, s, 6-OCH_3_), 3.78 (3H, s, 8-OCH_3_); ^13^C-NMR (125 MHz, DMSO-*d*_6_, δ) 175.7 (C-4), 162.0 (C-7), 160.3 (C-2), 150.9 (C-4′), 147.5 (C-5), 147.1 (C-9), 143.5 (C-6), 137.7 (C-8), 127.7 (C-2′, 6′), 123.0 (C-1′), 114.6 (C-10), 114.2 (C-3′, 5′), 106.0 (C-3), 61.8 (5, 7-OCH_3_), 61.4 (4′-OCH_3_), 61.3 (8-OCH_3_), 55.5 (6-OCH_3_). The structures of the seven compounds are shown in Figure 1. From the HPLC analysis of the ethyl acetate extract of *C*. *aurantium* dried immature fruits (Figure 2), naringin and neohesperidin, are responsible for predominant peaks. 

### 2.3. Contribution of the Identified Compounds to XO Inhibitory Activity

**Figure 3:**
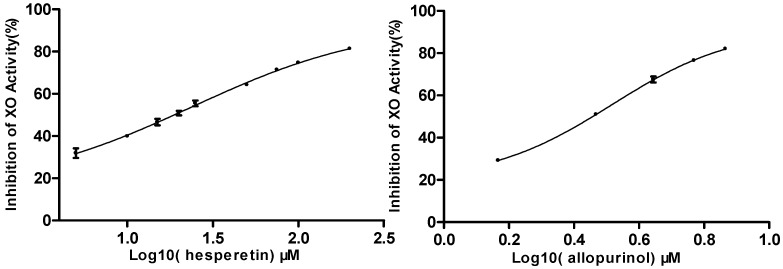
Concentration-dependent inhibition of XO activity by hesperetin and allopurinol. Data represent mean ± standard deviation (S.D.) of triplicate experiments.

Seven identified compounds were evaluated for their ability to inhibit the production of uric acid from xanthine by xanthine oxidase. An overview about the effects of these substances on XO activity is given in Table 2. Neohesperidin, hesperidin, naringin, and tangeretin showed weak inhibitory activities, while hesperetin, nobiletin, and naringenin displayed either potent or moderate activities at 200 μM with inhibition rates of 81.3%, 59.4%, and 49.8%, respectively. As shown in Figure 3, micromolar concentrations of hesperetin elicited dose-dependent inhibition of xanthine oxidase with an IC_50_ value of 16.48 μM, comparable to that 2.07 μM of the positive control allopurinol, a drug clinically prescribed in clinic for gout treatment. Naringenin, having a hydroxyl substituent at C-4′ in ring C, displayed lower activity than hesperetin, with a methoxyl group at C-4′ and a hydroxyl group at C-3′ in ring C. These results are in agreement with the previous report on their XO inhibitory activities [25]. Previous studies have demonstrated that the substitution of the hydroxyl group at C-7 of the basic flavonoid structure by a glycoside seems to decrease the XO inhibitory effect [8]. Therefore, the presence of the glycosides are responsible for the lower inhibitory activities of neohesperidin, hesperidin, and naringin.

**Figure 4:**
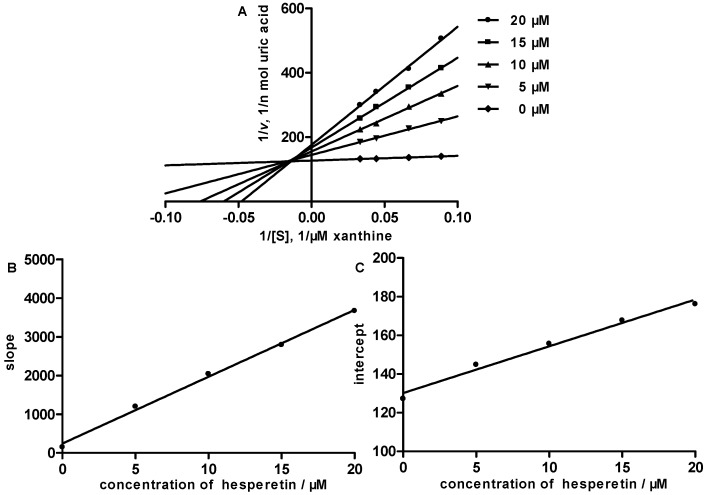
Inhibition kinetics of xanthine oxidase by hesperetin. (**A**) Lineweaver-Burk plots in the absence or at the different concentrations of hesperetin. Each point is the average value from triplicate experiments. Secondary plots to calculate the inhibition constants are shown in (**B**) (K_i_) and (**C**) (K_I_).

To understand the enzyme inhibition mode of hesperetin, the Lineweaver-Burk plots were established, as shown in Figure 4. Since the Lineweaver-Burk plots of hesperetin cross to the left of the 1/V axis but above the 1/[S] axis, it belongs to the mixed type of inhibition. In mixed inhibition, the inhibitor can bind to the free enzyme as well as to the enzyme-substrate complex. The inhibitor constant Ki, the dissociation constant of the enzyme of the-inhibitor complex, can be calculated from the slope of the inhibited curve, and the inhibitor constant K_I_, the dissociation constant of the enzyme-substrate-inhibitor complex, can be calculated from the y-intercept of the inhibited curve. The K_i_ and K_I_ of hesperetin were determined to be 1.40 μM and 53.85 μM, respectively. Parallel studies were carried with allopurinol, the data indicate that the mode of inhibition by allopurinol is of the competitive type with a K_i_ of 1.92 μM.

For the first time, nobiletin and tangeretin, the main polymethoxyflavones in the ethyl acetate extract of *C*. *aurantium* immature fruits, were tested for their ability to inhibit the production of uric acid from xanthine by xanthine oxidase exhibiting only moderate or weak effects. Moreover, pharmacokinetic investigations have demonstrated that neohesperidin and hesperidin are transformed into hesperetin by human intestinal bacteria [26,27]. These data strongly indicate that the actual *in vivo* effect of ethyl acetate extract from *C*. *aurantium* dried immature fruits is considerably stronger than indicated by the present *in vitro* experiment. Further *in vivo* research is being carried out to identify a potential inhibition for complementary use in the prevention and treatment of hyperuricemia.

## 3. Materials and Methods

### 3.1. Chemicals

Analytical grade petroleum ether, ethyl acetate, *n*-butanol, methanol, ethanol, dimethyl sulphoxide, and hydrochloric acid were obtained from Beijing Chemical Industry Group Co., Ltd, (Beijing, China). Methanol and acetonitrile employed for high-performance liquid chromatography (HPLC) were HPLC super gradient quality (Tedia Company Inc., Fairfield, OH, USA). Allopurinol, XO, and xanthine were purchased from Sigma-Aldrich Chemicals (St. Louis, MO, USA).

### 3.2. General Experimental Procedures

A Bruker AV-500 FT-NMR spectrometer was used, operating at 500.1 MHz for ^1^H and at 125.8 MHz for ^13^C, chemical shifts are expressed in δ referring to the residual solvent signals δ_H_ 2.50 and δ_C_ 39.5 for DMSO-*d*_6_, coupling constants, *J*, are in hertz. ESI-MS was acquired with a Bruck micro-TOFQ mass spectrometer (Bruck, Bremen, Germany). Column chromatography was performed over a RP-18 reversed-phase silica gel (S-50 μm; YMC, Kyoto, Japan). Thin-layer chromatography (TLC) analysis was carried out on a pre-coated TLC plate with silica gel RP-18 60 F254 (0.25 mm, Merck, Darmstadt, Germany). Detection was achieved by spraying the sample with 10% H_2_SO_4_ in MeOH followed by heating. Preparative HPLC was performed using a Shimadzu LC-6AD pump connected to a Shimadzu SPD-20A UV-VIS detector (at 254 nm) with a Shim Pak ODS column (250 mm × 21.2 mm, i.d., 10 μm, Shimadzu, Kyoto, Japan) and a Megress ODS column (250 mm × 20 mm, i.d., 10 μm, Jiangsu hanbon Science and Technology Co., Ltd., Huai′an, China). Analytical HPLC was performed with two LC-20AT solvent delivery pumps, an online mixer, a SIL-20A autosampler, a CTO-20A column temperature controller, and a SPD-M20A photodiode-array detector (Shimadzu). HPLC-grade water was purified using a Milli-Q system (Millipore, Boston, MA, USA). All solvents used for the chromatographic separations were distilled before use.

### 3.3. Plant Materials

Dry immature fruits of *Citrus aurantium* L., mature fruit pericarp of *Citrus* *medica* L., mature fruit of *Citrus* *medica* L. var. *sarcodactylis* Swingle, and immature fruit pericarp, mature fruit pericarp, and mature fruit exocarp of *Citrus reticulata* Blanco, sold commercially as medicinal herbs, were purchased from Zhewan Pharmaceutical Company, Bozhou, Anhui province of China, and identified by Prof. Baomin Feng, Dalian University, China. Voucher specimens for each herb (2014AFI-01, 2014CF-01, 2014CSF-01, 2014CRPV-01, 2014CRP-01, and 2014CER-01, respectively) were deposited at the School of Pharmacy, Qingdao University, China.

### 3.4. Extraction and HPLC Analysis of the Extracts

Four sets of each medicinal herb (2 kg) were reflux extracted separately twice with petroleum ether, ethyl acetate, butanol, and ethanol–water (75:25, *v/v*). The resultant extracts were then filtered, concentrated under reduced pressure, and evaporated to dryness and the percentage yield (*w/w*) of the extracts are given in Table 1. HPLC analysis of the extracts was performed as follows. 64 mg of each extract was dissolved in a mixture of methanol and water (1:1, *v/v*, 2 mL) and filtered with a membrane filter (Jinteng Nylon66, 0.45 μm, Tianjin Jinteng Experimental Equipment Co., Ltd., Tianjin, China), and 5 μL of the solution was subjected to HPLC under the following conditions. The chromatographic separation was carried out on a XDB-C18 (250 mm × 4.6 mm, 5 µm, Agilent Technologies, Inc., Santa Clara, CA, USA). The mobile phase consisted of acetonitrile (A) and water containing 2% acetic acid (B). A gradient program was used: 0–30 min, linear change from A–B (10:90, *v/v*) to A–B (20:80, *v/v*); 30–50 min, linear change from A–B (20:80, *v/v*) to A–B (40:60, *v/v*); 50–60 min, linear change from A–B (40:60, *v/v*) to A–B (60:40, *v/v*); and 60–70 min, isocratic elution with A–B (60:40, *v/v*). The flow rate was 1.0 mL/min, and the column temperature was maintained at 25 °C. The product was detected by monitoring UV absorption (SPD-M20A, Shimadzu) at 280 nm.

### 3.5. XO Inhibitory Activity Assay

All the extracts, fractions and isolated compounds were assayed for their XO inhibitory activity. The assay was performed according to the method modified by our group [13], based on the procedure reported by Masuda *et al.* [28]. The assay mixture consisting of 200 μL of test solution of various concentration, 140 μL of 70 mM phosphate buffer (pH = 7.5), and 120 μL enzyme solution (0.02 units/mL in the same buffer) was prepared immediately before use. After pre-incubation at 25 °C for 15 min, the reaction was initiated by the addition of 240 μL of substrate solution (300 μM xanthine in the same buffer). The assay mixture was incubated at 25 °C for 30 min. The reaction was stopped by adding 100 μL of 1 M HCl, and uric acid level was determined by an HPLC method using a Diamonsil ODS-C18 column (250 × 4.6 mm i.d., 5 μm, Dikma Technologies, Beijing, China) on an Agilent 1260 system equipped with a G1311C quaternary pump, a G1329B autosampler, a G1316A thermostatted column compartment, and a G1314F variable wavelength detector coupled with an analytical workstation (Agilent Technologies, Inc.). After filtration, 20 μL of the sample was injected into the column and eluted with 100 mM sodium dihydrogen phosphate in water at a flow rate of 1 mL/min. The eluate was monitored for absorbance at 290 nm. All assays were conducted in triplicate, thus inhibition percentages are the mean of triplicate observations. Inhibition percent was calculated by the following equation: inhibition (%) = [(peak area of uric acid in control experiment) – (peak area of uric acid in sample experiment)]/(peak area of uric acid in control experiment) × 100. The IC_50_ values of the active compounds were calculated from regression lines of a plot of the percentage of inhibition on XO activity *versus* the concentrations of the samples. Different concentration of the tested samples were dissolved in dimethyl sulphoxide (DMSO) and, subsequently, diluted with 70 mM phosphate buffer (pH = 7.5) to a final concentration containing less than 1% DMSO (*v/v*), which did not affect the enzyme assay. Allopurinol, a known inhibitor of XO, was used as the positive control.

### 3.6. XO Inhibitory Modes of Action Assay

Enzyme kinetics were determined in the absence and presence of the tested samples with varying concentrations of xanthine (37.5, 50, 75, and 100 μM) as the substrate, where tested samples were at various concentration (2–20 μM) and xanthine at a certain concentration, using the XO assay methodology. The plots were drawn by the reciprocal velocity (ν-1) *versus* the concentration of the substrate (1/[xanthine]).

### 3.7. Isolation and Identification of Compounds

The ethyl acetate extract of *C*. *aurantium* dried immature fruits (20 g), which showed the highest XO inhibitory activity, was subjected to RP-18 reversed-phase silica gel column eluted with a gradient increasing MeOH (20%–100%) in water to give six subfractions (Fr. 1, 4.3 g; Fr. 2, 3.7 g; Fr. 3, 1.5 g; Fr. 4, 2.6 g; Fr. 5, 3.9 g; Fr. 6, 3.2 g) on the basis of TLC analyses. Fr.2 was purified by preparative HPLC using MeOH–H_2_O (40:60) at a flow rate of 2.0 mL/min (Megres C_18_ column, 250 mm × 20 mm, i.d., 10 μm) to yield compound **1** (428.5 mg, t_R_ = 71 min), **2** (51.4 mg, t_R_ = 87 min), and **3** (97.5 mg, t_R_ = 102 min). Compound **4** (65.3 mg, t_R_ = 85 min) and **5** (71.8 mg, t_R_ = 112 min) were obtained from Fr. 4 by preparative HPLC (Shim Pak ODS column, 250 mm × 21.2 mm, i.d., 10 μm; flow rate, 3.0 mL/min) employing MeOH–H_2_O (60:40) as the mobile phase. Fr. 5 was isolated by preparative HPLC using MeOH–H_2_O (80:20) at a flow rate of 2.8 mL/min (Megres C_18_ column, 250 mm × 20 mm, i.d., 10 μm) to yield compound **6** (40.2 mg, t_R_ = 62 min) and **7** (33.3 mg, t_R_ = 78 min). Their structures were elucidated on the basis of spectroscopic methods, including MS, 1-D, and 2-D NMR spectral techniques.

### 3.8. Statistical Analysis

Data from the XO inhibitory assay were expressed as the mean ± standard deviation (S.D.) and were analyzed by one-way analysis of variance using the SPSS version 13.0 software (SPSS, Inc., Chicago, IL, USA). A value of *p* < 0.05 was considered statistically significant. IC_50_ values of the samples were calculated with origin version 8.0 software (OriginLab Corporation, Northampton, UK). The inhibitory type and *K*_i_ value were analyzed using GraphPad Prism 5.0 software (GraphPad Software, Inc., La Jolla, CA, USA).

## 4. Conclusions

In summary, 24 organic extracts of four species belonging to *Citrus* genus recording in the Chinese Pharmacopeia were prepared and their XO inhibitory activities evaluated in order to develop therapeutic or preventive agents for hyperuricemia. The results demonstrated for the first time that the ethyl acetate extract of *C*. *aurantium* dried immature fruits possessed XO inhibitory activity. Bioguided fractionation by chromatography of the extract to obtain five flavanones and two polymethoxyflavones showed that hesperetin was a putative candidate for the XO inhibitory activity.

## Figures and Tables

**Table 1:** Yield and XO inhibitory effects of the prepared extracts.

Species	Parts	Solvent	Yield (*w/w* %)	XO Inhibition (Mean ± S.D.%)
				200 μg/mL
*Citrus aurantium* L.	Immature fruits	A	0.08	31.64 ± 0.25
		B	2.55	60.70 ± 0.74
		C	11.45	46.25 ± 0.27
		D	21.01	37.26 ± 0.35
*Citrus medica* L.	Mature fruit pericarp	A	0.28	27.63 ± 0.75
		B	4.52	19.08 ± 0.07
		C	13.87	-
		D	16.69	-
*Citrus medica* L. var. *sarcodactylis* Swingle	Mature fruit	A	0.33	21.38 ± 0.99
		B	2.05	23.36 ± 0.53
		C	5.33	17.60 ± 0.05
		D	20.08	12.85 ± 0.48
*Citrus reticulata* Blanco	Immature fruits pericarp	A	0.27	39.76 ± 0.31
		B	2.47	50.15 ± 0.44
		C	8.00	38.73 ± 0.24
		D	12.95	29.01 ± 1.11
*Citrus reticulata* Blanco	Mature fruit pericarp	A	0.22	41.46 ± 0.25
		B	1.78	33.11 ± 0.70
		C	17.68	12.32 ± 1.29
		D	23.89	-
*Citrus reticulata* Blanco	Mature fruit exocarp	A	0.45	26.44 ± 0.56
		B	3.80	23.41 ± 0.88
		C	15.97	10.90 ± 0.44
		D	18.34	-

**Table 2:** Xanthine oxidase inhibitory activities of the isolated compounds.

	Compounds	XO Inhibition (Mean ± S.D.%, 200 μM)	IC_50_ (μM)	Inhibition Mode	K_i_ (μM)
1	Naringin	15.29 ± 0.64			
2	Hesperidin	7.28 ± 0.21			
3	Neohesperidin	5.36 ± 0.01			
4	Naringenin	49.82 ± 1.17			
5	Hesperetin	81.31 ± 0.36	16.48	mixed	1.40
6	Nobiletin	59.44 ± 0.83	107.53	noncompetitive	77.24
7	Tangeretin	28.82 ± 0.02			
	Allopurinol		2.07	competitive	1.92

## References

[1] Richette P., Bardin T. (2010). Gout. Lancet.

[2] Hsieh J.F., Wu S.H., Yang Y.L., Choog K.F., Chen S.T. (2007). The screen and characterization of 6-aminopurine-based xanthine oxidase inhibitors. Bioorg. Med. Chem..

[3] Terkeltaub R.A. (2003). Clinical practice. Gout. N. Engl. J. Med..

[4] Becker M.A., Kisicki J., Khosravan R., Wu J., Mulford D., Hunt B., MacDonald P., Joseph-Ridge N. (2004). Febuxostat (TMX-67), a novel, non-purine, selective inhibitor of xanthine oxidase, is safe and decreases serum urate in healthy volunteers. Nucleos. Nucleot. Nucl..

[5] Fukunari A., Okamato K., Nishino T., Eger B.T., Pai E.F., Kamezawa M., Yamada I., Kato N. (2004). Y-700 [1-[3-cyano-4-(2,2-dimethylpropoxy)phenyl]-1*H*-pyrazole-4-carboxylic acid]: A potent xanthine oxidoreductase inhibitor with hepatic excretion. J. Pharmacol. Exp. Ther..

[6] Pacher P., Nivorozhkin A., Szabo C. (2006). Therapeutic effects of xanthine oxidase inhibitor: Renaissance half a century after the discovery of allopurinol. Pharmacol. Rev..

[7] Nguyen M.T.T., Awale S., Tezuka Y., Ueda J.Y., Tran Q.L., Kadota S. (2006). Xanthine oxidase inhibitors from the flowers of *Chrysanthemum sinense*. Planta Med..

[8] Spanou C., Veskoukis A.S., Kerasioti T., Kontou M., Angelis A., Aligiannis N., Skaltsounis A.L., Kouretas D. (2012). Flavonoid glycosides isolated from unique legume plant extracts as novel inhibitors of xanthine oxidase. PLoS ONE.

[9] Nile S.H., Park S.W. (2014). Antioxidant, α-glucosidase and xanthine oxidase inhibitory activity of bioactive compounds from maize (*Zea mays* L.). Chem. Biol. Drug Des..

[10] Liu H.X., He M.T., Tan H.B., Gu W., Yang S.X., Wang Y.H., Li L., Long C.L. (2015). Xanthine oxidase inhibitors isolated from *Piper nudibaccatum*. Phytochem. Lett..

[11] Luo Y.L., Qu W., Liang J.Y. (2013). Progress on chemical constituents and biological activities of the genus *Citrus*. Strait Pharm. J..

[12] Mencherini T., Campone L., Piccinelli A.L., Mesa M.G., Sánchez D.M., Aquino R.P., Rastrelli L. (2013). HPLC-PDA-MS and NMR characterization of a hydroalcoholic extract of *Citrus aurantium* L. var. *amara* Peel with antiedematogenic activity. J. Agric. Food Chem..

[13] Huo L.N., Wang W., Zhang C.Y., Shi H.B., Liu Y., Liu X.H., Guo B.H., Zhao D.M., Gao H. (2015). Bioassy-guided isolation and identification of xanthine oxidase inhibitory constituents from the leaves of *Perilla frutescens*. Molecules.

[14] Li X.C., Liu X.H., Gao H., Fan M.L., Liu K., Wang W. (2015). Study on inhibition and enzyme kinetics of different solvent extractions from *Polygonum cuspidatum* on xanthine oxidase. China Pharm..

[15] Lin J.K., Chen P.C., Ho C.T., Lin-Shiau S.Y. (2010). Inhibition of xanthine oxidase and suppression of intracellular reactive oxygen species in HL-60 cells by theaflavin-3,3′-digallate, (−)-epigallocatechin-3-gallate, and propyl gallate. J. Agric. Food Chem..

[16] Casquete R., Castro S.M., Martín A., Ruiz-Moyano S., Saraiva J.A., Córdoba M.G., Teixeira P. (2015). Evaluation of the effect of high pressure on total phenolic content, antioxidant and antimicrobial activity of citrus peels. Innov. Food Sci. Emerg..

[17] Mehmood B., Dar K.K., Ali S., Awan U.A., Nayyer A.Q., Ghous T., Andleeb S. (2015). *In vitro* assessment of antioxidant, antibacterial and phytochemical analysis of *Citrus sinensis*. Pak. J. Pharm. Sci..

[18] Sharma S., Kori S., Parmar A. (2015). Surfactant mediated extraction of total phenolic content (TPC) and antioxidants from fruits juices. Food Chem..

[19] Maltese F., Erkelens C., Kooy F., Choi Y.H., Verpoorte R. (2009). Identification of natural epimeric flavanone glycosides by NMR spectroscopy. Food Chem..

[20] Lei H.M., Sun W.J., Lin W.H. (2000). Studies on chemical constituents of *Citrus grandis* Osbecks. Var. *tomentosa* Hort. Northwest Pharm. J..

[21] Guo X.L., Wang T.J., Guo M.J., Chen Y. (2000). Studies on chemical constituents of processed green tangerine peel. Chin. J. Chin. Mater. Med..

[22] Feng F., Wang X.N., Yan C.M. (2012). Studies on chemical constituents of *Citrus aurantium* L.. Asia-Pac. Tradit. Med..

[23] Chen X.X., Shimayi R.H.M., Long S.J. (2013). Studies on chemical constituents of *Toddalia asiatica* stems. Northwest Pharm. J..

[24] Han S., Kim H.M., Lee J.M., Mok S.Y., Lee S. (2010). Isolation and identification of polymethoxyflavones from the hybrid *Citrus*, Hallabong. J. Agric. Food Chem..

[25] Dew T.P., Day A.J., Morgan M.R.A. (2005). Xanthine oxidase activity *in vitro*: Effects of food extracts and components. J. Agric. Food Chem..

[26] Kim D.H., Jung E.A., Sohng I.S., Han J.A., Kim T.H., Han M.J. (1998). Intestinal bacterial metabolism of flavonoids and its relation to some biological activities. Arch. Pharm. Res..

[27] Zhang W., Jiang S., Qian D.W., Shang E.X., Duan J.A. (2014). Determination of metabolism of neohesperidin by human intestinal bacteria by UPLC-Q-TOF/MS. Chromatographia.

[28] Masuda A., Takahashi C., Inai M., Miura Y., Masuda T. (2013). Chemical evidence for potent xanthine oxidase inhibitory activity of *Glechoma hederacea* var. *grandis* leaves (Kakidoushi-Cha). J. Nutr. Sci. Vitaminol..

